# Genome sequence of *Microbacterium* sp., an endophyte isolated from *Zea mays* in South Africa

**DOI:** 10.1128/mra.00881-26

**Published:** 2026-10-26

**Authors:** Beatrix Coetzee, Teresa A. Coutinho, Maffa O. Lekhetho, Yvonne Maila, Henry A. Njom

**Affiliations:** 1 Plant Protection Division, Agricultural Research Council, Infruitec-Nietvoorbij 111657 , Stellenbosch, South Africa; 2 Department of Biochemistry, Genetics and Microbiology, Centre for Microbial Ecology and Genomics/Forestry and Agricultural Biotechnology Institute, University of Pretoria 56410, Pretoria, South Africa; 3 Agricultural Research Council, Grain Crops 56397 , Potchefstroom, South Africa; University of Manitoba, Winnipeg, Canada

**Keywords:** *Microbacterium*, maize, genome sequencing, ARC56GC

## Abstract

*Microbacterium* species are plant-associated actinobacteria with plant growth-promoting potential. Here, the 3.42 Mb circular genome of strain ARC56GC isolated from maize leaves in South Africa is reported. Comparative genomics identified *Microbacterium testaceum* as its closest relative, while average nucleotide identity (ANI) analyses suggest that ARC56GC represents a genomically distinct lineage within the *M. testaceum* species complex.

## ANNOUNCEMENT

*Microbacterium* is a gram-positive actinobacterial genus associated with plants, and some members exhibit plant growth-promoting properties, including enhanced seedling growth and salinity tolerance in maize (1, 2). As part of a survey for potentially beneficial maize endophytes in South Africa, the genome of a maize-associated *Microbacterium* isolate was sequenced to expand genomic resources and provide a basis for future studies of its plant-associated functions.

Isolate ARC56GC was recovered from surface-disinfected symptomatic maize leaf tissue collected from a commercial field (GPS coordinates: 29.5500082, 30.2384825) in KwaZulu-Natal, South Africa, during the 2024/2025 growing season. Although the leaves exhibited symptoms consistent with Goss’ wilt, the study focused on recovering bacterial endophytes rather than investigating the disease. Leaf tissue was surface sterilized, macerated in sterile distilled water. Aliquots of the resulting suspension were streaked onto nutrient broth yeast extract (NBY) medium and incubated at 25–28°C for 3–5 days (3). A triple-purified isolate producing yellow-orange mucoid colonies was selected and confirmed as gram-positive by KOH testing and Gram staining. DNA from the culture was extracted using the Zymo Quick-DNA Fungal/Bacterial Miniprep Kit (Zymo Research, Irvine, CA) according to the manufacturer’s recommendations.

DNA was mechanically sheared using a Megaruptor 3 (Diagenode), and libraries were prepared using the PacBio HiFi Plex Prep and SMRTbell library preparation workflows according to the manufacturer’s protocols. Sequencing on the PacBio Revio platform using the Revio SPRQ Polymerase Kit and SMRT Cell 25M generated 8,287 HiFi reads with an *N*_50_ of 6,496 bp (NCBI SRA SRR39659930). *De novo* assembly with Flye v2.9.2 (4) produced a 3,416,393 bp circular genome with a guanine + cytosine (G + C) content of 69.64% and 14× genome coverage (NCBI accession JCASAO000000000). The genome was rotated in Geneious 9.1.8 (Biomatters, Ltd., Auckland, New Zealand) to position the *dnaA* gene at the beginning of the sequence. CheckM v1.0.18 (5) quality assessment in KBase (6) estimated 98.99% completeness and 0.51% contamination, indicating a high-quality genome assembly.

GTDB-Tk v2.3.2 (7) identified *Microbacteriaceae* bacterium M105 (GCA_022702225; *M. testaceum*, taxonomically inconclusive) as the closest reference genome, sharing 97.91% average nucleotide identity (ANI). Genome BLAST Distance Phylogeny (GBDP) and 16S rRNA gene phylogeny inferred using the Type (Strain) Genome Server (TYGS) (8) did not confidently assign ARC56GC to species level, although *Microbacterium testaceum* NBRC 12675 was identified as the closest type strain. Digital DNA–DNA hybridization (dDDH) values against *M. testaceum* NBRC 12675 were 76.2% (*d*_0_), 43.0% (*d*_4_), and 69.8% (*d*_6_), indicating substantial genomic relatedness but divergence across homologous regions (8, 9).

Pairwise ANI analysis using M1CR0B1AL1Z3R (10) revealed considerable intraspecific diversity within *M. testaceum* (ANI < 95). ARC56GC clustered most closely with *Microbacteriaceae* bacterium M105 (97.77%) and *M. testaceum* SORGH_AS_0426 (97.38%; GCF_030818605), both associated with cereal or grass hosts in the USA (11).

**Fig 1:**
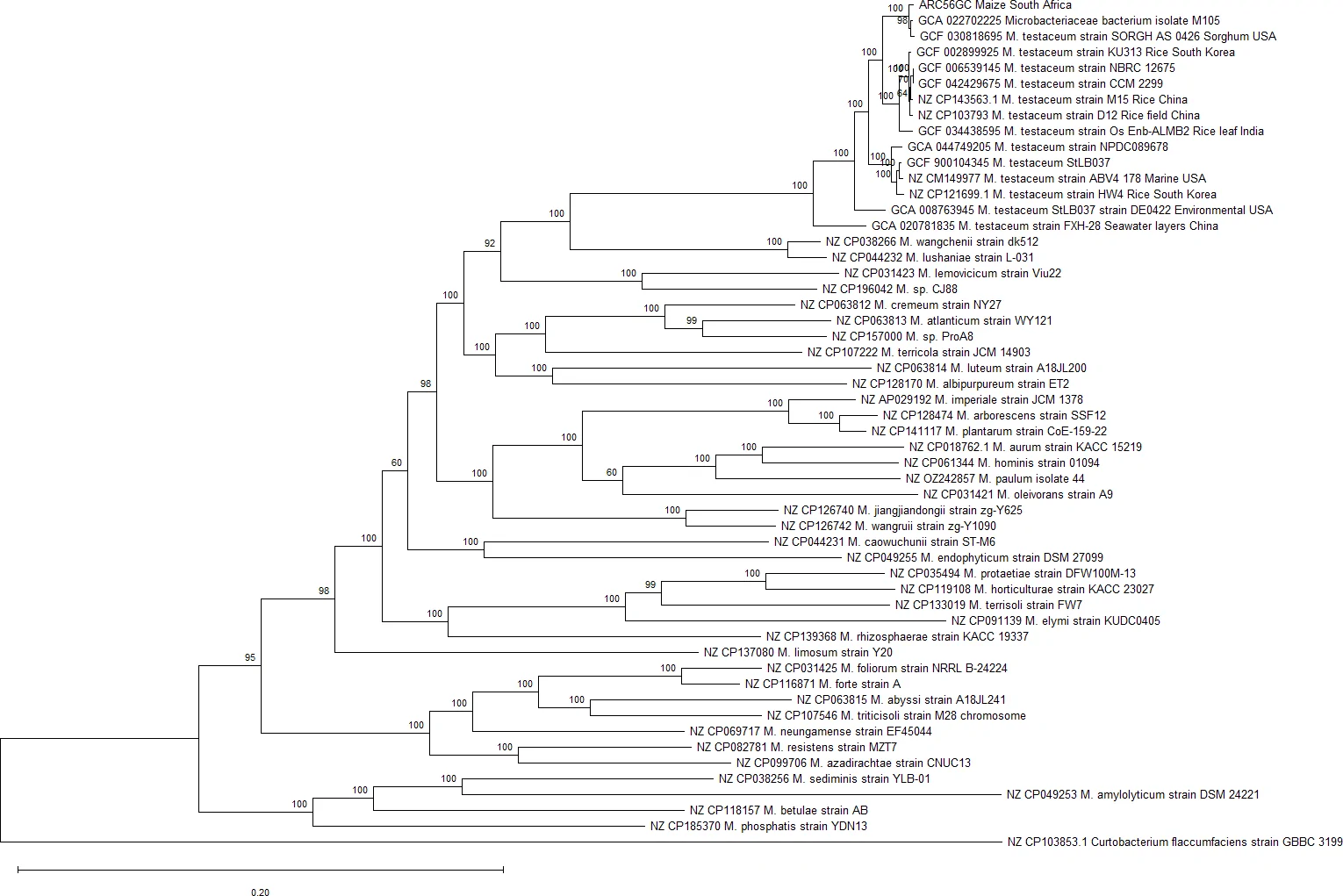
Maximum-likelihood phylogenomic tree showing the position of strain ARC56GC within the genus *Microbacterium*. The tree was reconstructed using the M1CR0B1AL1Z3R 2.0 web server from concatenated alignments of 173 core genes shared among the analyzed genomes (ortholog identification criteria: ≥70% amino acid identity and ≥70% alignment coverage). Core genes defined as genes present in all genomes included in the analysis represent the conserved core proteome and are used to infer the vertical evolutionary relationships among taxa. Numbers at branch nodes indicate bootstrap support values. Where available, the geographic origin and host or environmental source of isolation are indicated for *M. testaceum* strains.

At the genus level, core-genome phylogenetic analysis using M1CR0B1AL1Z3R (Fig. 1) was congruent with the ANI clustering, with ARC56GC consistently grouping with M105 and SORGH_AS_0426 (Tables S1 and S2 at https://doi.org/10.6084/m9.figshare.33093251 and Fig. S1 at https://doi.org/10.6084/m9.figshare.33093251). Together with the dDDH results, these findings indicate that ARC56GC forms a well-supported, genomically distinct lineage within the *M. testaceum* species complex.

## Data Availability

The PacBio HiFi sequencing reads and genome assembly generated in this study have been deposited in the NCBI database under BioProject PRJNA1490675. The raw PacBio HiFi reads are available in the Sequence Read Archive (SRA) under accession SRR39659930, and the genome assembly is available under NCBI accession JCASAO000000000. Supplemental Tables and Figures are available from Figshare at https://doi.org/10.6084/m9.figshare.33093251.
